# Systematic review to understand and improve care after stillbirth: a review of parents’ and healthcare professionals’ experiences

**DOI:** 10.1186/s12884-016-0806-2

**Published:** 2016-01-25

**Authors:** Alison Ellis, Caroline Chebsey, Claire Storey, Stephanie Bradley, Sue Jackson, Vicki Flenady, Alexander Heazell, Dimitrios Siassakos

**Affiliations:** Obstetrics and Gynaecology, Southmead Hospital, Westbury on Trym, Bristol, BS10 5NB UK; International Stillbirth Alliance, Bristol, UK; Southmead Hospital, Westbury on Trym, Bristol, BS10 5NB UK; University of Surrey, Surrey, UK; Mater Research Institute -The University of Queensland (MRI-UQ), Brisbane, Australia; Institute of Human Development, Faculty of Medical and Human Sciences, University of Manchester, Manchester, UK; St. Mary’s Hospital, Central Manchester University Hospitals NHS Foundation Trust, Manchester Academic Health Science Centre, Manchester, M13 9WL UK; University of Bristol, School of Social & Community Medicine, Bristol, UK

**Keywords:** Stillbirth, Bereavement, Care

## Abstract

**Background:**

2.7 million babies were stillborn in 2015 worldwide; behind these statistics lie the experiences of bereaved parents. The first Lancet series on stillbirth in 2011 described stillbirth as one of the “most shamefully neglected” areas of public health, recommended improving interaction between families and frontline caregivers and made a plea for increased investment in relevant research.

**Methods:**

A systematic review of qualitative, quantitative and mixed-method studies researching parents and healthcare professionals experiences of care after stillbirth in high-income westernised countries (Europe, North America, Australia and South Africa) was conducted. The review was designed to inform research, training and improve care for parents who experience stillbirth.

**Results:**

Four thousand four hundred eighty eight abstracts were identified; 52 studies were eligible for inclusion. Synthesis and quantitative aggregation (meta-summary) was used to extract findings and calculate frequency effect sizes (FES%) for each theme (shown in italics), a measure of the prevalence of that finding in the included studies.

Researchers’ areas of interest may influence reporting of findings in the literature and result in higher FES sizes, such as; *support memory making* (53 %) and *fathers have different needs* (18 %). Other parental findings were more unexpected; Parents want *increased public awareness* (20 %) and for *stillbirth care to be prioritised* (5 %).

Parental findings highlighted lessons for staff; *prepare parents for vaginal birth* (23 %), *discuss concerns* (13 %), *give options & time* (20 %), *privacy not abandonment* (30 %), *tailored post-mortem discussions* (20 %) and *post-natal information* (30 %).

Parental and staff findings were often related; *behaviours and actions of staff have a memorable impact on parents* (53 %) whilst staff described *emotional, knowledge and system-based barriers to providing effective care* (100 %). Parents reported distress being caused by midwives hiding behind ‘doing’ and ritualising guidelines whilst staff described distancing themselves from parents and focusing on tasks as coping strategies.

Parents and staff both identified the need for improved *training* (parents 25 % & staff 57 %); *continuity of care* (parents 15 % & staff 36 %); *supportive systems & structures* (parents 50 %); and c*lear care pathways* (parents 5 %).

**Conclusions:**

Parents’ and healthcare workers’ experiences of stillbirth can inform training, improve the provision of care and highlight areas for future research.

**Electronic supplementary material:**

The online version of this article (doi:10.1186/s12884-016-0806-2) contains supplementary material, which is available to authorized users.

## Background

2.7 million babies were stillborn in 2015 worldwide [1]. In the UK, 3286 babies were stillborn in 2013 [2]; approximately 10 bereaved families every day.

The experiences of bereaved parents were recognised in a series of papers in the Lancet as key to bringing about change [3]. The 2011 series identified stillbirth as one of the “most shamefully neglected” areas of public health and recommended improving interactions between families and frontline caregivers [4], and made a plea for increased investment in relevant research [5]. In 2016, the Lancet Ending Preventable Stillbirth Series addresses progress against the goals laid out in 2011 [6]. The series emphasised the enduring economic, psychological and social costs of stillbirth which need to be addressed, not only by stillbirth prevention, but also by improvements in care for bereaved families. In 2014, the World Health Organization released a statement on preventing and eliminating disrespect and abuse during facilty-based childbirth [7]. This respectful and comprehensive care should include the newborn [8] and not end with death; dignified maternal and newborn care matters to grieving parents [9].

Critically, the provision of care for families when a child is stillborn is vitally important to prevent short and long-term negative outcomes [10]. Current care for bereaved parents after a baby dies is inconsistent [11, 12], and parents are more likely to develop prolonged psychological problems if professional support is not given [13]. Bereaved parents have been identified as a high-risk group for complicated grief [14], with up to 25 % suffering severe symptoms years after the death of their baby [15] . The support received by the mother following the death of her child was the single most important factor in predicting the nature of the grief process that she would experience [16].

Midwives find caring for bereaved families stressful and emotionally challenging [17], with many experiencing difficulty with this area of practice [18], feeling unprepared due to a lack of support and training [19, 20]. While women and their families interpret the experience of stillbirth as the birth and death of a baby and a major family tragedy, hospital staff appear to view it as a clinical problem. This mismatch of focusing on ‘clinical’ rather than ‘personal’ care appeared to cause distress to parents [21].

Despite the impact stillbirth has on both parents and staff, it remains an area in which most obstetricians and midwives receive little or no training [22, 23], and 31 % of those who received training said it was inadequate [24]. A survey of over 2000 UK staff found that one third of respondents reported unsatisfactory training to counsel parents about investigations after stillbirth [25].

In 2009, the Cochrane Collaboration published a systematic review of the support available for parents and their families following perinatal death [26]. Of the three trials identified for potential inclusion in the review all were excluded because of the high loss-to-follow-up rate. Therefore, no judgment could be made on the advantages or disadvantages of certain behaviours or management currently used in bereavement care. As a result, the review authors recommended other study designs should be used to inform practice.

No previous research has systematically analysed the available evidence on parents’ views on the experience of going through a stillbirth, or key healthcare workers experiences of caring for couples dealing with a stillbirth. This systematic review aimed to assess the current available evidence, extract findings and highlight key themes that may help to guide midwifery and medical management, training of key healthcare workers and development of support services dealing with bereaved parents in the future.

## Methods

### Objective

The objective was to review and meta-summarise studies of parents’ and healthcare workers’ experience of maternity bereavement care for stillbirth, in Western High-Income-Country hospital settings, with the aim of developing practical learning points that can be applied to clinical training for healthcare workers.

### Design

The study was designed by a multi-professional research team, with experience in stillbirth research, to inform research, training, and ultimately improve care for parents who experience late (≥24 weeks gestation) intrauterine fetal death (stillbirth).

Synthesis and quantitative aggregation (meta-summary) of qualitative data [27] was used as it allows aggregation and interpretation of studies that may be excluded from Cochrane reviews.

### Search strategy

Search terms were formulated using the SPIDER Framework [28]. Due to poor systematic indexing of qualitative research on many databases, all synonyms and North American variations for stillbirth were searched, alongside thesaurus terms on databases, to improve the sensitivity of the search strategy (Additional file 1).

The databases searched included; AMED, EMBASE, MEDLINE, Psych INFO, BNI and CINAHL. Databases were searched up to March 2014 by a medical librarian (SB). The initial search took place in November 2013 with monthly automated search updates via NICE evidence. Conference abstracts from the International Stillbirth Alliance and First Candle conferences have also been hand-searched for eligibility.

### Eligibility criteria

The inclusion and exclusion criteria were set to optimise the number of relevant studies chosen for inclusion. Qualitative, quantitative and mixed-method studies that assessed parents’ or healthcare workers experience of care after stillbirth were included. The meta-summary focused on Western High-Income Countries; European, North American, Australian and South African studies were included. Studies from other settings were excluded as healthcare provision and cultural and religious practices are likely to be sufficiently different to render aggregation with Western studies inadvisable. In relation to this, studies not available in English were excluded. As international definitions of stillbirth vary and many papers researched a combination of fetal loss types it was difficult to set a gestational age for study exclusion without losing a large amount of relevant data therefore, only studies exclusively addressing miscarriage, fetal loss before 24 weeks, lethal fetal diagnosis or neonatal death were excluded. Studies addressing views of indirect family members were excluded. The words *coil, device* and *mirena* were set as exclusion criteria to reduce inappropriate cross-referencing with the shortened term for intrauterine device (IUD). No date limitations were set. All relevant studies with original data, from ethnography to large surveys, were eligible for inclusion. Conference abstracts that contained extractable findings were included. Review articles, dissertations and books were excluded.

### Study selection

Combining search results provided an initial screen. Six investigators (AE, CC, CS, SB, SJ and DS) excluded studies by screening abstracts. Reasons for exclusion included: duplicates; topic not relevant to stillbirth care; topic not relevant to parent or staff experience; location of study non-western or low-middle income country; not available in English; review articles; dissertations. Disagreements on whether to include or exclude studies were discussed by the research team to reach a consensus. This generated a list of potential full text articles which were obtained (SB) and assessed independently by two investigators (AE and CC). Full text articles were excluded using the same criteria, with the addition of; no relevant findings found in data.

### Data extraction

Data extraction was performed by two investigators (AE and CC). A data extraction form was developed to help standardize and log the data extraction process (Additional file 2). In the validation phase, two investigators (AE and CC) completed the data extraction form for the same three papers and inter-rater reliability was concluded by consensus of the research team (AE, CC, CS, SB, SJ and DS). In the definitive phase two investigators (AE and CC) independently extracted findings from relevant papers.

### Data analysis

Data analysis was based on the meta-summary approach, a quantitative aggregation of qualitative findings, developed by Sandelowski [27]. The method comprises: (a) extraction of relevant statements of *findings* from each report; (b) reduction of these statements into abstracted findings; and, (c) calculation of effect sizes. The primary research team (AE, CC, CS and DS) reviewed and discussed all of the extracted findings. With consensus the research team summarized the findings into thematic sentences. The thematic sentences were developed with clinicians in mind and written as learning points for healthcare professionals to help them improve care for bereaved parents.

In the method an inter-study matrix organizes reports by the abstracted findings, and an intra-study matrix organizes the findings by the reports, thereby allowing the same information to be seen in different ways. The inter-study matrix is used to calculate the frequency effect size (FES) of each abstracted finding, which is defined as the number of reports containing the finding divided by the total number of reports [27]. The intra-study matrix is used to calculate the intensity effect size (IES), which is defined as the number of findings produced in each study divided by the total number of findings across all studies [27]. The calculation of effect sizes is a way to unite the empirical precision of quantitative research with the descriptive precision of qualitative research. The calculation of effect sizes constitutes a quantitative transformation of qualitative data to extract more meaning, verify the presence of patterns or themes across studies, and add a quantitative ‘weight’ to each finding and to each study. This method facilitates the interpretation and usability of the results for healthcare professionals. FES and IES were calculated for parent and staff studies separately.

## Results and discussion

### Study selection

**Fig. 1:**
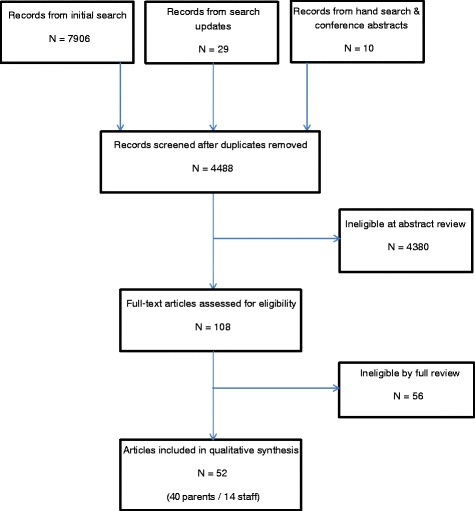
PRISMA flow diagram. Legend: 52 studies eligible for inclusion; 40 parent studies and 14 staff studies (2 studies addressed both parents + staff)

The initial search strategy identified 7906 abstracts. An additional 29 abstracts were identified by database updates and 10 conference abstracts were identified by hand search. After duplication and eligibility screening 108 were selected for full text extraction. Of the 108 full-text papers reviewed 52 were eligible for inclusion (Fig. 1).

### Findings

**Fig. 2:**
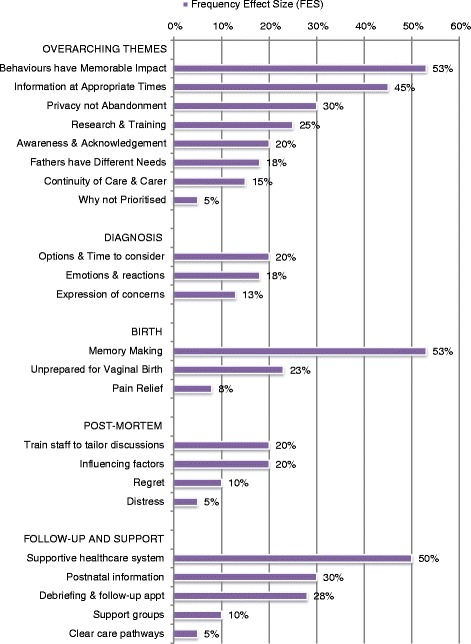
Chart of parent thematic sentence frequency effect sizes

**Fig. 3:**
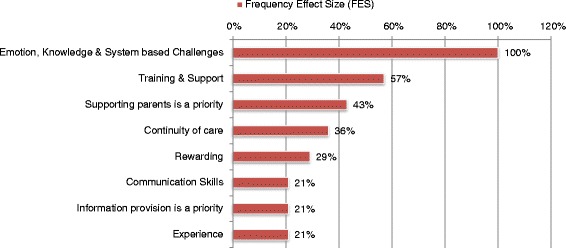
Chart of staff thematic sentence frequency effect sizes

Of the 52 studies included, 40 articles related to parents’ experiences and 14 to healthcare workers experiences of stillbirth care. The majority of papers reported questionnaire, interview and focus group studies, analyzed using a variety of qualitative methods and statistics. A few personal accounts were eligible for inclusion; study size therefore, varied from 1 to 2631 participants. Articles were published over an 18-year period from 1996 to 2013. Studies were conducted in nine countries. Parent studies were conducted across eight countries (Table 1) and Staff studies were conducted across five countries (Table 2). Overall 623 individual findings were extracted from the studies. Twenty-three parent themes with thematic sentences were identified and used for calculation of FES (Table 3; Fig. 2) and IES (Additional file 3). Eight staff themes with thematic sentences were identified and used for calculation of FES (Table 4; Fig. 3) and IES (Additional file 4). Tables 3 and 4 present a representative selection of the extracted findings which were used by the research team to develop the thematic sentences; these tables help to describe the thematic sentences in more depth. A summary of each included study can be found in Additional files 3 and 4.

**Table 1:** Location of parent studies

Location	Number of Studies
Australia	4
Canada	1
Norway	2
South Africa	1
Sweden	15
Republic of Ireland	2
United States of America	8
United Kingdom	7

**Table 2:** Location of Staff studies

Location	Number of studies
Australia	2
Italy	1
Republic of Ireland	3
United States of America	3
United Kingdom	5

**Table 3:** Parent thematic sentences

Parents thematic sentences	FES	Sample quotes from extracted findings
Overarching Themes		
1. Behaviours and actions of staff can have a memorable impact on parents	53 %	"A detached attitude from staff was identified as being particularly unhelpful" (Dyson 1998) [32]
		"It was reported that verbal communication with staff ceased during the examination, but that body language of staff showed that something was wrong with the fetus. This silence worries women, who said that it would have been better if the staff had talked during the examination – explaining what they saw or what was puzzling them. Almost all participants thought that the examination room had been full of personnel who were communicating with each other without involving the parents in the discussion" (Trulsson 2004) [48]
		"It is apparent that training medical personnel in the emotional and caring aspect of ‘breaking bad news’ during routine scans needs to be re-examined" (McCreight 2008) [38]
		"When mothers experienced that they did not get the care they needed from the hospital staff, they felt neglected by them" (Erlandsson, Lindgren 2011) [33]
		"Death notification was also particularly difficult and mothers perceived professionals to be uncertain, avoidant, and tenuous" (Nordlund 2012) [39]
		"Parents perceiving communication positively reported far more simple statements with intensifiers, such as ‘I’m so sorry’, and non-verbal expressions of sympathy, such as personal touch and the health care professional expressing emotion" (Pullen 2012) [41]
		"Midwives hiding behind ‘doing and ritualizing the guidelines was unhelpful" (Dyson 1998) [32]
		"Regardless of the experience, the women wrote in glowing terms about the respect, kindness and professionalism of healthcare providers during and immediately after labour" (Lee 2012) [21]
		"Several issues caused particular distress. The woman generally attributed such issues to the hospital policy and procedure, the broader system, rather than to any inadequacy of individual health care providers" (Lee 2012) [21]
		"Health professionals also supported motherhood through seeing the mother and father as the parents of their child" (Radestad 2011) [41]
		"Mothers felt that their emotional states were disregarded when the professionals primary focus was on practical matters such as death reporting or funeral planning" (Nordlund 2012) [39]
		"Mothers felt ignored in the corridors, noting that health professionals often avoided making eye contact" (Nordlund 2012) [39]
		"Some of the mothers reported that professionals did not listen well and lacked empathy" (Nordlund 2012) [39]
		"Most meaningful for all parents were the physicians and nurses who took time to sit with them, look them in the eye, and be present with them in their sadness" (Kelley 2012) [37]
		"Where this physicians instincts or training may have led him to attempt to protect the patient from his emotional reaction, the patient instead found the reaction to be deeply human and a sign of shared grief over a terrible loss" (Kelley 2012) [37]
		"Fathers felt their fatherhood went unrecognized or invalidated" (Cacciatore 2012)
		"Asking the parents to look at the monitor as non-verbal reinforcement of verbal communication of death" (Pullen 2012) [41]
		"When describing what was positive with the way they were informed synonyms with honesty/clarity and empathy/intimacy were most frequently reported. In contrast, lack of eye contact, empathy and hesitations from healthcare professionals in confirming the baby’s death were described as negative experiences" (Gavensteen 2013)
2. Clear, easily understandable and structured information given sensitively at appropriate times, helps parents through their experience	45 %	"Being informed about what happens next was something all the mothers described as important and as having been insufficient after the diagnosis" (Malm 2011) [51]
		"Parents refused to see their babies because they feared the baby would be deformed or monstrous. Their fears were not spoken out loud so were not countered by information from healthcare workers" (Malacrida 1997) [50]
		"Parents appreciated a sensitive description of how the baby might look" (Dyson 1998) [32]
		"Initially reluctant to see their babies because they knew they would not look normal" (Lee 2012) [21]
		"Sometimes it was difficult for the fathers to understand the information given to them, especially if the staff used professional terms" (Samuelsson 2001) [47]
		"When the baby was born the women experienced total silence. For a few this was expected, but for most it came as a shock" (Trulsson 2004) [48]
		"Most parents thought that their child should be delivered by caesarean section. They needed information, advice and support every step of the way for a normal delivery" (Saflund 2004) [46]
		"Waiting without knowing for what or for how long" (Malm 2011) [51]
3. Parents want privacy not abandonment	30 %	"The intense grief mothers were already suffering was exacerbated by hearing the cries of other newborns in the ward and seeing pregnant women" (Norlund 2012)
		"Several mothers reported that hearing noises of the bustling activity and other births around them added to their suffering" (Kelley 2012) [37]
		"Parents who reported negative communication reported the healthcare professional leaving the room immediately after diagnosis. Positively reported when healthcare professionals stayed with them for a while before leaving" (Pullen 2012) [41]
4. Research and multiprofessional training is important for all staff to improve standards of bereavement care	25 %	"Hospital staff must be better trained in the significance of the loss of a baby and in how to help bereaved mothers" (Conry 2008) [31]
		"Parents feel that more funding and research focus should be directed at preventing stillbirth" (Wildsmith 2008) [55]
		"Noticed a significant gap in knowledge and comfort level with perinatal loss and bereavement by health care professionals outside the labour and delivery programme" (Forhan 2010) [54]
		"Many parents suggested healthcare providers be given special training in communicating after a stillbirth" (Flenady 2010) [53]
5. Parents wish for increased awareness and acknowledgement of stillbirth	20 %	"Society at large does not understand the profundity and significance of the loss of a baby" (Conry 2008) [31]
		"Antenatal classes should include, as part of their syllabus, a discussion on the loss of a baby, what parents should do in such circumstances and the grief process" (Conry 2008) [31]
		"Parents struggle with the silence and taboo that surrounds stillbirth" (Kelley 2012) [37]
		"Much of the isolation is caused by the awkwardness and discomfort felt by others " (Kelley 2012) [37]
		"Lack of understanding or support from family and friends" (Kelley 2012) [37]
		"Stillborn child is real and will always be remembered as part of their family" (Kelley 2012) [37]
		"Identity is not recognized by others" (Kelley 2012) [37]
6. Fathers may have different needs to mothers; they want to be involved in decision making and often focus on practical tasks	18 %	"Fathers recommended that the staff should not forget the fathers, even though, for obvious reasons all the attention is focused on the child in the search for signs of life. Fathers also recommended that staff should break the frightening silence before and in connection with the revelation of the death of the baby, should not use medico-technical terms, and should speak with both parents at the time. They should acknowledge the expression of grief and give a respectful response" (Samuelsson 2001) [47]
		"The father has a special need for information and participation before, during and after delivery of a stillborn child" (Samuelsson 2001) [47]
		"Frustration and helplessness may well be inevitable, since the father cannot shield his partner from pain" (Samuelsson 2001) [47]
		"Fathers often kept their emotions under control for fear of upsetting the mother" (Kavanaugh 2005) [36]
		"Mothers wanted fathers to express their emotions" (Kavanaugh 2005) [36]
		"Fathers were unsure of how to support the mother" (Kavanaugh 2005) [36]
7. Continuity of care and carer is important to parents	15 %	"All women said that it would have given them a great sense of security if they had met the same caregivers at induction of labour as those on the day the stillbirth was diagnosed" (Trulsson 2004) [48]
		"They might not meet the physician again due to routine care" (Erlandsson 2011) [34]
		"Negative lack of continuity of care versus positive presence of continuity of care" (Pullen 2012) [41]
8. Parents with a baby who died in-utero may feel that their care is not appropriately prioritised by staff	5 %	"Some mothers waited extended periods of time for a physician to confirm the baby’s death, and others felt that they did not receive appropriate medical care from professionals because their baby was dead" (Nordlund 2012) [39]
		"Women thought they were not given priority or that they were not considered important once their baby was dead. They felt the information they received was insufficient, they had to change room several times, they saw several different doctors, and several midwives were involved in their delivery" (Trulsson 2004) [48]
**Diagnosis**		
9. To be involved in decision making parents appreciate being given options and the time to consider them	20 %	"Lack of information is perceived to be an obstacle to the mothers participation" (Malm 2011) [51]
		"They were left with little time for preparation or discussion" (Malacrida 1997) [50]
		"The physicians, while informing the mothers about the alternatives, tried to make them responsible for their own birth process" (Erlandsson 2011) [34]
		"Mothers should be acknowledged and allowed to take part in decision making" (Erlandsson 2011) [34]
10. Parents have a range of emotions and reactions because stillbirth is a life changing event	18 %	"Shock, a feeling of being paralysed, speechlessness, a lack of feeling, escape, and denial. The situation was nightmarish and indescribable, and the fathers found it difficult to comprehend what had happened" (Samuelsson 2001) [47]
		"The women described the time immediately after they learned their baby was dead as unreal and numbing. They were in turmoil with feelings of anger and sorrow. They had difficulty comprehending what had happened and what it all meant" (Trulsson 2004) [48]
		"Failure by medical staff to convey reassurance, they also overlook, and so fail to acknowledge, emotional aspects of the loss" (McCreight 2008) [38]
11. Staff should support parents to express their concerns	13 %	"Parents describe how their complaints and symptoms were not taken seriously by healthcare professionals" (Malacrida 1997) [50]
		"Entertained some hope on arrival in the maternity ward" (Samuelsson 2001) [47]
		"A fear that a dead baby would be the cause of their partners falling sick was obvious for some" (Samuelsson 2001) [47]
		"Almost all women had a premonition that something was wrong with their baby before they contacted the hospital, including symptoms such as less or absent fetal movement, and a feeling of heaviness in the abdomen" (Trulsson 2004) [48]
		"Women who reported they had difficulty communicating their worry did not want to be viewed as troublesome or unnecessarily worried" (Trulsson 2004) [48]
		"Many suspected something was wrong with their unborn baby prior to diagnosis. Most frequently they had reduced or no fetal movements" (Gavensteen 2013)
**Birth**		
12. Spending time and making memories with their baby should be an option that is supported and offered more than once	53 %	"Parents received the general message that you should say goodbye, but there are strict limits on where you can do it and how long it should take" (Malacrida 1997) [50]
		"Time in which the baby’s identity as an individual and a human being was defined" (Lee 2012) [21]
		"Identifying the baby as part of the family" (Lee 2012) [21]
		"She didn’t realize that she could hold her baby, no-one said that it was OK to do" (Kerslake 2012) [58]
		"Striking a balance between not pressing some women too hard and gently leading other women who need more support to cope with such contact is indeed a challenge" (Radestad 2001) [42]
		"Mothers of stillborn babies felt more natural, good, comfortable and less frightened if the staff supported assumptive bonding by simply offering the baby to the mother" (Erlandsson 2013) [49]
13. Support and Information from staff may help parents who feel emotionally unprepared for a vaginal birth	23 %	"Waiting for the induction was difficult for the women" (Trulsson 2004) [48]
		"Being offered delivery options or having explanation of why the options were limited was positively reported" (Pullen 2012) [41]
		"Women commented that they did not expect to have to go through labour in the normal way" (Dyson 1998) [32]
		"(Vaginal birth) Made the baby ‘more real’" (Lee 2012) [21]
		"Pleased in retrospect (re. vaginal birth)" (Lee 2012) [21]
		"They all believed that the natural procedure conveyed more dignity" (Samuelsson 2001) [47]
		"Frustration and helplessness may well be inevitable, since the father cannot shield his partner from pain" (Samuelsson 2001) [47]
		"They all immediately perceived the plan as appalling they saw no meaning in giving birth to a dead baby" (Trulsson 2004) [48]
		"Once the idea of giving birth normally had taken hold, women accepted it, and their focus shifted to going through childbirth" (Trulsson 2004) [48]
		"Once accomplished giving birth…they felt fortified by this event" (Trulsson 2004) [48]
		"Going through labour seemed a ‘sick way of hurting her’" (Kerslake 2012) [58]
		"Most parents thought that their child should be delivered be caesarean section. They needed information, advice and support every step of the way for a normal delivery" (Saflund 2004) [46]
14. Pain relief options should be fully discussed with parents	8 %	"Mothers who were heavily sedated during their labour had profound regrets at the lost opportunity to be with their baby" (Malacrida 1997) [50]
**Post-Mortem**		
15. Parents want improved training so that staff can provide tailored discussions and written information to help them make informed decisions about Post-Mortem and funeral arrangements	20 %	"The option of limited PM was not discussed with parents who refused a full PM" (Yee Khong 1997)
		"Thought that they had not been counselled about the advantages of PM, even though many had additional counselling" (Yee Khong 1997)
		"It seemed particularly important that the opportunity of time was given for discussion and questions, and parents welcomed discussions specific to their situation, such as being told that PM might be useful in their circumstances" (Breeze 2012) [52]
		"Parents who needed less knowledge declined PM…if clinicians believed that such parents would benefit from information gained from a PM, then counselling needs to be more specifically targeted to address underlying attitudes" (Breeze 2012) [52]
		"All parents agreed written information about an autopsy, as well as that given verbally, was important" (Flenady 2010) [53]
16. There are many factors which influence parents decision whether to have a Post-Mortem	20 %	"Professional advise affected parents decisions to have an autopsy" (Heazell 2012) [25]
		"Parents frequently depend on others for decision making, either involving family and friends to help interpret information or placing responsibility on healthcare professionals to make the decision on their behalf" (Flenady 2010) [53]
17. Parents may regret certain decisions made regarding Post-Mortem and funeral arrangements	10 %	"Many parents expressed regret and guilt over leaving their children's bodies for the hospitals to dispose of and for deciding against a funeral" (Malacrida 1997) [50]
		"Parents who did not receive a death certificate expressed regret. The legal documentation legitimised their loss" (Malacrida 1997) [50]
		"All parents who did not have an autopsy expressed some regret or doubt about their decisions. No parent who had an autopsy expressed these feelings" (Flenady 2010) [53]
		"Where an autopsy was performed, none of these women wished that it had not been carried out" (Gavensteen 2013)
18. Long delays and inconclusive results can cause distress to parents	5 %	"A number of parents awaiting post-mortem results were very frustrated with the delay in receiving the results" (Kavanaugh 2005) [36]
		"Parents who experienced stillbirth wanted to understand the cause of their child's death and found it frustrating when no answers could be given" (Kelley 2012) [37]
**Follow-up and Support**		
19. Parents would appreciate a healthcare system ready to provide emotional support following birth and discharge from hospital	50 %	"Parents perceived lack of contact as an indicator that they should get on with recovery quickly and quietly" (Malacrida 1997) [50]
		"These women experience stillbirth not as a medical problem or temporary interruption in their reproductive lives, but as the birth and death of a baby" (Lee 2012) [21]
		"Fathers looked on themselves as a buffer between the world outside and their partner, and tried to spare her by taking care of practical matters themselves" (Samuelsson 2001) [47]
		"Hospitals should consider employing or contracting grief experts to help them deal with bereaved mothers" (Conry 2008) [31]
		"Feelings that caregivers had abandoned them when staff did not offer their services in bereavement" (Saflund 2004) [46]
		"Being warm and empathetic not mechanical and cursory" (Saflund 2004) [46]
		"Many of the parents comments focused on well-meaning but hurtful comments from clinicians who meant to be supportive but simply did not know what to say or do to offer comfort after a stillbirth…the most common and most hurtful comments were reassurances that they would have another baby" (Kelley 2012) [37]
		"There is no substitute for a dead child" (Kelley 2012) [37]
20. Parents should be supplied with information about what to expect post-natally	30 %	"Women felt that they should have been prepared for the post-partum symptoms" (Malacrida 1997) [50]
		"Many parents did not realize that they were entitled to maternity leave or pay" (Malacrida 1997) [50]
		"Different needs and reactions could sometimes cause misunderstandings (between mothers and fathers)" (Samuelsson 2001) [47]
		"Fathers found that it was usually more difficult for men to talk about grief and feelings that it was for women" (Samuelsson 2001) [47]
21. A debriefing and follow-up appointment can help resolve uncertainty	28 %	"Parents often blame their actions for the stillbirth and feel guilty. Parents need to be reassured that they did not cause the stillbirth" (Malacrida 1997) [50]
		"To meet the physician and midwife involved after the stillbirth and have the opportunity to talk about the event was beneficial for the grieving process" (Saflund 2004) [46]
		"Offer of special antenatal care for the next pregnancy eased their despair" (Saflund 2004) [46]
		"Option to meet the same caregivers in the next pregnancy increased the parents sense of security" (Saflund 2004) [46]
22. Support groups are helpful for many parents	10 %	"Support groups, or talking with other bereaved parents, was reported in being the most helpful thing in dealing with the death of their child" (Cacciatore 2007) [66]
		"Mothers were particularly helped by feeling that they were not alone in their grief, and that someone else could understand the experience" (Cacciatore, Bushfield 2007) [29]
23. Clear care pathways are required at the interface between primary and secondary care	5 %	"Hospitals should have a more structured procedure in place for dealing with bereaved mothers" (Conry 2008) [31]

**Table 4:** Staff thematic sentences

Staff thematic sentence	FES	Sample quotes from extracted findings
1. There are challenges that may prevent staff from providing effective bereavement care; Emotion, Knowledge and System based	100 %	"Midwives found it challenging and ‘emotionally draining’ to deal with their own ‘shock’ and ‘confusion’ at having to provide perinatal loss care…at times they felt ‘uncomfortable’ providing perinatal loss care. Providing perinatal loss care had the potential to become ‘all consuming and exhausting’" (Fenwick 2007) [20]
		"Feelings of inadequacy" (Fenwick 2007) [20]
		"There was ample time to talk with these women but many staff still avoided them, with the excuse that there was ‘no time’" (Begley 2003) [67]
		"Postpartum support was perceived as the hardest situation" (Rivaldi 2010) [75]
		"Some nurses tried to distance themselves in order to provide care" (Puia 2013) [74]
		"Nurses expressed the sentiment of holding it together until later" (Puia 2013) [74]
		"Shaken to the core; physically the nurses reported feeling stressed, muscle tension, headache, and pressure. Other nurses described difficulties eating and sleeping. Other nurses had consuming thoughts while awake" (Puia 2013) [74]
		"Never forget; holding onto grief. The nurses all noted that they could still vividly remember the fetal deaths (Puia 2013) [74]
		"Admitted to fear of further upsetting grief stricken parents" (Downe 2012) [70]
		"A number of students expressed the view that they did not receive enough education on this topic before being faced with a situation on the wards. Because the students didn’t feel confident in this area the students often tried to avoid women who had lost babies" (Begley 2003) [67]
		"The physicians in the focus groups would not routinely offer an autopsy to the parents, but would conduct one if requested" (Kelley 2012) [37]
		"Stillbirth has become a protocol driven tick box exercise" (Curtis 2000) [69]
2. Staff want improved training and a supportive working environment	57 %	"None of the students sourced professional support readily available to them through their university" (McKenna 2011) [19]
		"Clinicians discussed the importance of improving education on the data surrounding the prevalence of stillbirth and causes" (Kelley 2012) [37]
		"General agreement that targeted training and support were required to ensure the essential processes are effectively undertaken, and that empathetic attitudes are developed" (Downe 2012) [70]
		"There was evidence of positive innovation and good practice" (Downe 2012) [70]
3. Emotional support and acknowledging the birth and death of a baby is an important part of bereavement care	43 %	"Role recognition – midwives describe a generalized role in bereavement support with a consensus that ‘being there’ may be more important than doing" (Nallen 2006) [73]
		"Several of the physicians were less inclined to encourage mothers or parents to talk about their feelings unless initiated by the patient, out of concern that they may make the parents feel worse" (Kelley 2012) [37]
4. Continuity of care is important to staff	36 %	"Providing continuity of care was important" (Fenwick 2007) [20]
		"Rapport, friendships and close emotional bonds…essential in providing satisfying quality of care" (Fenwick 2007) [20]
		"Meet the individual needs of the women" (Fenwick 2007) [20]
		"Participants frequently raised the importance of liaison between midwives, health visitors and general practitioners as being crucial to the ability to provide quality continuing care for bereaved parents" (Cartwright 2005) [68]
5. Caring for bereaved parents can be rewarding for staff	29 %	"Being able to share this experience was viewed as an important positive was to honour and respect the baby’s life and existence" (Fenwick 2007) [20]
		"Receiving ‘positive’ feedback was important and provided midwives with a sense of achievement" (Fenwick 2007) [20]
6. Verbal and non-verbal communication skills are important	21 %	"Particular emphasis was placed on knowing what to say, when to say it and knowing when was inappropriate" (Nallen 2006) [73]
		"Listening was identified as an important communication skill" (Nallen 2006) [73]
		"All physicians acknowledged that they struggle with what to say and what not to say following a stillbirth" (Kelley 2012) [37]
7. Providing parents with information, enabling them to be actively involved in decision making, is a staff priority	21 %	"While midwives recognized that women and partners were often in a state of shock they also acknowledged that it was important for them to be active participants in what was happening to them" (Fenwick 2007) [20]
		"Midwives considered sharing information and knowledge with families a way of relieving anxiety and assisting women to ‘gain control’" (Fenwick 2007) [20]
8. Experience and knowledge may ease the provision of bereavement care but can increase the emotional burden felt by staff	21 %	"Midwives refer to experience as a valuable commodity and a resource that should be used to ease a woman's distress when she gives birth to a stillborn baby" (Curtis 2000) [69]
		"participants developed significantly from their initial experiences to positions where they reported such situations as rewarding and feeling they had something substantial to offer" (McKenna 2011) [19]
		"If physicians rated their training as comprehensive, and as self-rated performance increased, they were more likely to experience symptoms of depression" (Farrow 2013) [71]

Parent themes were catagorised by the area of care that they related to; *Diagnosis, Birth, Post-mortem*, or labeled as *Overarching* if reported in more than one of these areas. Staff themes were analysed and presented separately.

### Overarching thematic sentences

1. Behaviours and actions of staff can have a memorable impact on parents, FES 53 % [21, 29–48] – All verbal and non-verbal communication with and around parents can have a memorable impact. Hospital policy, procedures and systems can cause distress to parents. See Table 5 for a completed list of positive and negative behaviours and actions of staff, as reported by parents in the included papers.

**Table 5:** Behaviours & Actions of staff that can have a memorable impact

Positive Behaviours & Actions	Negative Behaviours & Actions
Explanation of staff actions - especially in advance of & during ultrasound scanning	Use of euphemisms
Simple statements with intensifiers - such as ‘I’m so sorry’	Silence without explanation around diagnosis
Non-verbal expressions of sympathy - touch	Topic avoidance
Non-verbal reinforcement of diagnosis - such as	Negative body language - lack of eye contact, hesitation
showing the ultrasound screen to parents	Detached attitude from staff
Honesty	Ritualization of guidelines
Clarity	Hiding behind ‘doing’ jobs
Letting parents express themselves	Staff communicating without involving parents in discussions
Significant others presence for support	Reassurance they would have another baby
Staff expressing emotions	
Understanding & empathic staff	
Emotionally astute & instinctual staff	
Respect	
Kindness	
Professionalism	
Individualising care	
Acknowledging & supporting parental roles	
Listening to parents	
Simply spending time with parents	
Supporting parents to grieve
2. Clear, easily understandable and structured information given sensitively at appropriate times, helps parents through their experience, FES 45 % [21, 31, 32, 34, 36, 37, 39–42, 44–51] – Parents describe insufficient information provision from diagnosis through to the birth of their baby. Acknowledgment of the event and parent’s concerns is imperative for holistic care. Individualised information, delivered sensitively and in the right pace for parents can help them through their experience.
3. Parents want privacy not abandonment, FES 30 % [21, 31, 33–35, 37, 39, 41, 42, 46, 47, 51] – Parents wanted the space to support each other in private but positively reported healthcare professionals who spent time with them. Seeing and hearing other mothers and babies added to parents suffering.
4. Research and multiprofessional training is important for all staff to improve standards of bereavement care, FES 25 % [31, 35, 37, 38, 40, 52–56] **–** Parents feel that there is a significant gap in healthcare professionals’ knowledge and comfort level dealing with perinatal loss and bereavement after stillbirth. All healthcare professionals who have contact with parents, within the hospital and community, should receive special training to improve care.
5. Parents wish for increased awareness and acknowledgement of stillbirth, FES 20 % [21, 31, 37, 38, 47, 50, 57, 58] **–** Stillbirth is a taboo subject for society. Parents feel isolated by the lack of support and understanding that they receive. Parents want increased awareness and recognition of stillbirth.
6. Fathers may have different needs to mothers; they want to be involved in decision making and often focus on practical tasks, FES 18 % [30, 33, 34, 36, 37, 40, 47] **–** Fathers have a special need for information and participation. Fathers can find it difficult to express themselves. They want to protect and support their partner but feel frustrated and helpless if they cannot do this.
7. Continuity of care and carer is important to parents, FES 15 % [34, 37, 40, 41, 46, 48] **–** Parents appreciated and feel reassured by meeting with familiar staff throughout their care.
8. Parents with a baby who died in-utero may feel that their care is not appropriately prioritised by staff, FES 5 % [39, 48] – Parents felt they were not considered important once their baby had died. Waiting to see professionals, having multiple carers, changing rooms and receiving insufficient information made parents feel their care was not a priority to staff.

### Diagnosis thematic sentences

9. To be involved in decision making parents appreciate being given options and the time to consider them, FES 20 % [38, 41–43, 47, 50, 51, 59] **–** Giving parents information and allowing them the time to consider the options empowers parents and enables them to be involved with decision making. Lack of information is perceived as an obstacle to parents’ participation and control.
10. Parents have a range of emotions and reactions because stillbirth is a life changing event, FES 18 % [33, 36, 37, 46–48] **–** Parents reactions to the diagnosis of stillbirth are unique. Parents describe a diverse range of emotions from sorrow to anger and denial. Parents commonly found it difficult to comprehend what had happened. Healthcare professionals need to be aware of this normal range of responses, allow parents to express their feelings and support them through this time.
11. Staff should support parents to express their concerns, FES 13 % [35, 41, 47, 48, 50] – Many parents suspected something was wrong with their baby prior to presentation and described how their symptoms were not always taken seriously by healthcare professionals. Parents reported difficulty communicating their worry as they did not want to be viewed as troublesome.

### Birth thematic sentences

12. Spending time and making memories with their baby should be an option that is supported and offered more than once, FES 53 % [21, 29, 31, 32, 35–37, 39, 40, 42–44, 46–50, 58–61] – Parents who decide not to see and hold their baby often later regret this decision. Parents should be made aware of this and offered the opportunity more than once. Parents’ choices should be supported by staff and the time spent with their baby should be led by the parents.
13. Support and information from staff may help parents who feel emotionally unprepared for a vaginal birth, FES 23 % [21, 32, 33, 37, 46–48, 51, 58] **–** Many parents did not expect to have to go through labour and vaginal birth, their immediate supposition was that their baby would be delivered by caesarean section. It is important to explain the options and the reasons why one of the options may be better in their individual circumstances. For vaginal birth parents need information, advice and support at every step. In retrospect, women reported being empowered by a vaginal birth.
14. Pain relief options should be fully discussed with parents, FES 8 % [31, 47, 50] – Advantages and disadvantages of pain relief options should be fully discussed. Some mothers who were sedated held regrets about the lost opportunity to be with their baby.

### Post-mortem thematic sentences

15. Parents want improved training so that staff can provide tailored discussions and written information to help them make informed decisions about post-mortem and funeral arrangements, FES 20 % [25, 35–37, 40, 52, 53, 56] **–** Staff should be trained to discuss information regarding post-mortem and funeral arrangement options with parents in a clear and empathic manner. Parents appreciate information tailored to their individual circumstances and supplementary written information to help them make an informed decision.
16. There are many factors which influence parents decision whether to have a post-mortem, FES 20 % [25, 35, 38, 46, 52, 53, 56, 62] **–** There are many reasons why parents choose to have a post-mortem or not. See Table 6 for a completed list of the reasons reported by parents in the included papers. The barriers perceived by staff are often different than those reported by parents.

**Table 6:** Factors that influence parents decision whether to have a post-mortem

Influencing Factors
Shock and grief hinder decision making
Perception of invasiveness of autopsy
Explanation
Healthcare professionals
Family members
Cause of death
Transferring baby
Time to get results
Recurrence of risk
Effect on next pregnancy
Confirmation of diagnosis at pre-natal investigations
Altruism
17. Parents may regret certain decisions made regarding post-mortem and funeral arrangements, FES 10 % [31, 35, 50, 53] **–** Parents who decline post-mortem regret their decision more commonly than parents who accept post-mortem. Parents may also regret not arranging their own funeral.
18. Long delays and inconclusive results can cause distress to parents, FES 5 % [36, 37] **–** A lack of communication about timescales and the meaning of post-mortem results may further parents distress.

### Follow-up and support thematic sentences

19. Parents would appreciate a healthcare system ready to provide emotional support following birth and discharge from hospital, FES 50 % [21, 29, 31–38, 42, 43, 45–47, 50, 59, 63–65] **–** Parents experience stillbirth not as a medical problem, but as the birth and death of a baby. Parents perceived a lack of contact in the post-natal period as an indicator that they should get on with recovery quickly and quietly. Hospitals should consider developing specialist bereavement support services and employing specifically trained staff.
20. Parents should be supplied with information about what to expect post-natally, FES 30 % [31, 34–38, 40, 47, 50, 51, 61, 64] **–** Differing needs and reactions often cause misunderstanding between parents. Parents feel unprepared for the postnatal period and are unaware of their entitlements and the services available. See Table 7 for a completed list of the post-natal information that parents in the included papers recommended should be discussed.

**Table 7:** Information about what to expect postnatally should include

Postnatal information
ᅟPhysical symptoms - lactation, after pains, bleeding
ᅟPsychological symptoms - depression, post-natal depression, anxiety, post-traumatic stress disorder
ᅟRisk factors for psychological problems after stillbirth
ᅟHow men’s and women’s emotional reactions often differ
ᅟFinancial considerations and entitlements - maternity pay, maternity leave
ᅟSupport groups & services
ᅟStillbirth certificates & registration
ᅟPlan for results & follow-up
21. A debriefing and follow-up appointment can help resolve uncertainty, FES 28 % [29, 31, 32, 35, 37, 39, 42, 46, 47, 50, 51] **–** A well-executed follow-up and debriefing appointment is beneficial to the parents grieving process. Good preparation and structure can aid this. See Table 8 for a completed list of parents recommendations for the debriefing and follow-up appointment from the included papers.

**Table 8:** Debriefing and Follow-up appointment

Debriefing and Follow-up appointment
ᅟShould occur in a timely fashion
ᅟLed by someone experienced in bereavement
ᅟContinuity is useful – doctor/midwife that have met the family before
ᅟUse of baby’s name
ᅟCause of stillbirth should be discussed
ᅟReassurance that it was not the parents fault is important
ᅟAcknowledgement and apologies should be made if there were mistakes
ᅟThe primary focus should be on the stillborn pregnancy, but future pregnancy should still be discussed including; psychological impact, stress of next pregnancy, antenatal care in future pregnancies
22. Support groups are helpful for many parents, FES 10 % [29, 37, 38, 66] – Talking with other bereaved parents, feeling that they are not alone, can help parents to deal with the death of their baby.
23. Clear care pathways are required at the interface between primary and secondary care, FES 5 % [31, 65] – Clear administrative processes should be in place for dealing with bereaved parents.

### Staff thematic sentences

1. There are challenges that may prevent staff from providing effective bereavement care; Emotion, Knowledge and System based, FES 100 % [19, 20, 25, 37, 67–76] **–** Caring for bereaved parents is challenging for staff. Healthcare professionals often lack confidence in their personal ability to provide good quality care for parents. Hospitals protocols and processes create barriers to providing holistic and individualised care. See Table 9 for a comprehensive list of the challenges staff reported in the included studies.

**Table 9:** Challenges that prevent staff from providing effective bereavement care

System based challenges	Emotion based challenges	Knowledge based challenges
Restricted time	Distress of parents	Answering parents questions
Too much paperwork	Unexpectedness	Unknown cause of stillbirth
Complexity and length of post-mortem consent form	Uncertainty	Lack of confidence
	Inadequacy	Lack of awareness of stillbirth rates
Level of support	Embarrassment	Lack of general understanding by staff
Providing privacy	Lack of confidence	Social and cultural differences
Labour ward floor plan	Personal opinions about investigations	
Lack of specialist staff	Personal distress	
	Emotionally over-whelmed	
	Trying to hold it together	
	Need for coping strategies i.e. avoidance	
	Difficulty concentrating	
	Difficulty coming back to work
2. Staff want improved training and a supportive working environment, FES 57 % [19, 20, 25, 37, 70, 74–76] – Targeted education and training would help to prepare staff and increase confidence in providing stillbirth care. Staff want a more supportive environment to help them deal with practical aspects and emotional impact of stillbirth.
3. Emotional support and acknowledging the birth and death of a baby is an important part of bereavement care, FES 43 % [20, 37, 67, 70, 73, 74] – Staff feel that it is important to support parents emotional reactions and grief responses, however many worry that encouraging parents to talk may make them feel worse.
4. Continuity of care is important to staff, FES 36 % [20, 68, 70, 73, 74] – Having time to spend with parents helps to enhance relationships and build rapport, which are essential for staff to feel that they are providing quality care however, staffing constraints can impede this.
5. Caring for bereaved parents can be rewarding for staff, FES 29 % [20, 67, 70, 74] – Utilising their skills to help parents deal with their grief is satisfying for staff.
6. Verbal and non-verbal communication skills are important, FES 21 % [20, 37, 73] – Following a stillbirth, staff struggle with not knowing what to say, or how and when to say it. Listening was identified as an important skill to staff.
7. Providing parents with information, enabling them to be actively involved in decision making, is a staff priority, FES 21 % [20, 70, 75] – Staff feel that sharing information and knowledge is a way of relieving anxiety, assisting parents to gain control and become active participants in decision making regarding their care.
8. Experience and knowledge may ease the provision of bereavement care but can increase the emotional burden felt by staff, FES 21 % [19, 69, 71] – Experienced staff feel that they have more to offer parents and can find caring for bereaved parents rewarding. Experience helps staff to provide support and information for parents however, experienced doctors may feel guilt and depression following a diagnosis of stillbirth.

## Discussion

### Main findings

This systematic review aimed to describe parents’ and health professionals’ views to help guide clinical management. We were able to identify inter-related experiences of parents and staff which can inform care delivery. Parents reporting dissatisfaction with the way the diagnosis was conveyed to them, not feeling involved with decision making, and not being given adequate time to come to terms with their loss and make decisions [11, 77, 78]. Critically, it was clear that staff actions and attitudes have a huge influence on parents’ decision making and ability to cope with the events [25, 77]. There is evidence that empathy and caring staff positively influence parents’ memories of their child [79] whilst a mismatch in parents’ and healthcare workers’ perception and management of stillbirth can cause long-lasting distress to parents [21]. Inadequate staff training was identified by staff and parents as impacting upon care provision for bereaved parents [22–25].

### Strengths and limitations

The main strength of this review is its inclusivity. The meta-summary method allows inclusion of studies of varied methodology that would not be considered for inclusion in Cochrane reviews. In addition, to capture data as widely as possible, we only excluded studies assessing the impact of miscarriage, termination of pregnancy and neonatal death alone. By limiting the location of the studies involved we also aimed to come up with a useful set of results and clinical recommendations applicable to high-income western settings, like our own. Although these restrict our findings, we believe that many of the experiences will be useful to also help improve care for parents bereaved through miscarriage, termination of pregnancy for fetal abnormality or the death of a baby after birth.

The main limitation is that this review was limited to high-income western studies. We recognise that access to healthcare and cultural differences in other countries may lead to different results and recommendations and therefore our findings need to be interpreted with caution in other locations. However, many of the findings fit within the WHO agenda for respectful care and so may still be applicable and adaptable for low-middle income settings [7]. We recognise that even within high-income western countries the results represent a generalisation of the key themes found in the literature and that personal experiences will vary therefore, it is important to always tailor care to individual parent’s circumstances.

High frequency effect sizes (FES) reflect high levels of reporting of these themes in the literature however, it is important to recognise that a high FES may reflect the focus of previous research over what is necessarily important to parents. We noticed that many of high FES themes, such as ‘*memory making’* and ‘*father’s needs’,* included findings from papers published by the same research team, who commonly have a particular research interest and publish multiple articles on similar topics. In contrast, many of the themes with low FES, such as ‘*public awareness’* and *‘prioritisation of stillbirth care’,* appeared to be coincidental findings, reported several times across multiple articles even though they were not usually directly related to the main aims of the studies. FES should therefore be interpreted with some caution, taken as a representation of the literature body rather than as a direct measure of importance to parents. In addition, themes with low FES may indicate areas of stillbirth care that require further research.

### Interpretation of results

There were some common themes that ran through the parent results, informing key lessons that can be learnt in the provision of care after stillbirth, from the time of diagnosis to hospital discharge and follow-up. Providing parents with understandable information, discussing options with them and tailoring care to their individual needs were common themes throughout the included studies. To achieve this knowledge-based and practical training are required to improve key healthcare workers ability and comfort providing personalised stillbirth care. Behaviours and actions of healthcare workers at all stages of care could leave a memorable effect on parents, including; ultrasonographers, general practitioners and health visitors as well as key maternity staff like midwives and obstetricians [38, 54]. This highlights the need for truly multi-disciplinary training to improve bereavement care for parents, crossing the primary and secondary care boundaries. Healthcare workers involved even prior to diagnosis could leave a memorable impact on parents [51, 69], so increased staff awareness of stillbirth is required to improve healthcare workers’ empathy towards parents’ pregnancy concerns.

We note that there were a limited number of articles on healthcare workers’ experiences eligible for inclusion and therefore, limited conclusions can be made. However, it was noticeable that there were a number of similarities and associations between parent and staff themes. Parents and staff both place similar importance on ‘*providing support’* and ‘*continuity of care’* to parents. Even when parent and staff findings did not directly mirror each other, associations could often be made. For example, staff described ‘*emotional, knowledge and system-based barriers to providing effective care’* which required often them to develop coping strategies such as distancing themselves from parents and focusing on tasks [74], whilst parents reported distress being caused by actions like midwives hiding behind ‘doing’ and ritualising guidelines [36], with these ‘*behaviours and actions of staff leaving a memorable impact on parents’*. Parents and staff also recommend similar improvements for training and service provision including; ‘*improved training’, ‘continuity of care’, ‘supportive systems and structures’,* and *‘clear care pathways’.*

There were some differences between parents’ and healthcare workers’ experiences. Noticeably parents’ reasoning for choosing whether or not to have a post-mortem was different to those expected by healthcare workers [25]. Importantly we need to recognise that parents reported that midwives commonly influenced their decision whether to have a post-mortem [25] and that parents who chose not to have a post-mortem were more likely to experience feelings of ‘*regret*’ [31, 35, 50, 53] therefore, it is important that all key healthcare workers are trained to provide information and ‘*tailored discussions’* around post-mortem decisions.

There were a number of findings that directly linked to service provision. Parents appreciated the availability of private rooms away from the noises and sights of the main maternity wards [39]. Hospitals should therefore consider developing a bereavement suite for parents experiencing a stillbirth. Parents also appreciated continuity of care, meeting the same staff members throughout their journey, which may have implications for staffing in hospitals. Specialist bereavement staff and clear care pathways also improved parents’ and healthcare workers’ experiences [31, 68]. Hospitals should therefore consider employing specialist bereavement staff to help support parents, act as a lead contact for parents awaiting follow-up and ensure care pathways are completed in a timely fashion. Alongside this training and support for other key multidisciplinary healthcare workers could help to improve parents’ experiences of care after stillbirth. Healthcare workers expressed the need for support during and after providing care for parents with stillbirth [20]. Even when formal services were available, they commonly were not used, with staff preferring to seek more informal support from their co-workers [19]. This mismatch needs to be recognised and time for informal support should be accommodated during work time.

Whilst there is a need for increased investment into research exploring the reasons for stillbirth and reducing stillbirth rates [5] the provision of care for families when such an event does occur remains vitally important to reduce the psychological impact on parents [10]. There is paucity of evidence that addresses the benefits and drawbacks of care provision and psychological interventions after fetal loss. A Cochrane review concluded there was insufficient evidence from randomised controlled trials to indicate whether or not there is benefit from interventions for families after perinatal loss [26]. In reality, randomised controlled trials which meet the inclusion criteria for the Cochrane review will be difficult to conduct, therefore developments in care will need to rely upon other forms of evidence. This systematic review has led to clinical and training recommendations that may improve care for bereaved parents but further research is needed to investigate these areas in more detail. Research is also needed in Low and Middle income countries where additional challenges for parents and healthcare workers are likely.

## Conclusion

Stillbirth has been recognised as one of the most neglected areas of public health. This systematic review highlights the importance of good quality bereavement care after stillbirth and the impact poor care can have on parents. Specific developments in training and service provision could help to improve care for bereaved parents, which may in turn improve psychological outcomes for parents. Further research is needed to assess the benefit and impact of these developments on parents.

## Additional files

Additional file 1:
**Search strategy.** (PDF 318 kb) Additional file 2:
**Data collection form.** (PDF 322 kb) Additional file 3:
**Parent Intensity Effect Size table, excel, description of individual articles used in the parent data set.** (XLSX 22 kb) Additional file 4:
**Staff Intensity Effect Size table, excel, description of individual articles used in the staff data set.** (XLSX 13 kb)
