# Supplementary material for: Systematic review to understand and improve care after stillbirth: a review of parents’ and healthcare professionals’ experiences
Source: BMC Pregnancy Childbirth. 2016 Jan 25;16:16. doi: 10.1186/s12884-016-0806-2 (PMC4727309; doi:10.1186/s12884-016-0806-2)
Supplement: Additional file 1: — Search strategy. (PDF 318 kb) [file 12884_2016_806_MOESM1_ESM.pdf]

Appendix 1: Search Strategy

Synonyms searched:

Sample	Mother, father, mum, dad, parent, couple, family, patient, woman, women, men, male, female, midwife, midwifery, nurse, doctor, medical, obstetrician, staff, healthcare worker
Phenomenon of Interest	Stillbirth, intrauterine death, intra-uterine death, in-utero death, IUD, IUFD, fetal loss, foetal loss, fetal death, foetal death, fetal demise, foetal demise, perinatal death
Design	All types of study will be included
Evaluation	Attitude, experience, perspective, opinion, view, theme, interview, focus group, questionnaire, survey
Research type	Qualitative studies, quantitative studies, and mixed method studies

MeSH headings searched by database:

Medline:

exp PARENTS/

FAMILY/

WOMEN/

MEN/

MIDWIFERY/

NURSES/

PHYSICIANS/

HEALTH PERSONNEL/

STILLBIRTH/

FETAL DEATH/

PERINATAL MORTALITY/

ATTITUDE/

INTERVIEW/

INTERVIEWS AS TOPIC/

QUESTIONNAIRES/

FOCUS GROUPS/

QUALITATIVE RESEARCH/

ABORTION, SPONTANEOUS/

EMBASE:

PARENT/

FATHER/

MOTHER/

FAMILY/

FEMALE/

MALE/

MIDWIFE/

NURSE/

PHYSICIAN/

HEALTH CARE PERSONNEL/

HEALTH PRACTITIONER/

STILLBIRTH/

FETUS DEATH/

PERINATAL MORTALITY/

ATTITUDE/

INTERVIEW/

QUESTIONNAIRE/

QUALITATIVE RESEARCH/

SPONTANEOUS ABORTION/

CINAHL:

exp PARENTS/

FAMILY/

WOMEN/

MEN/

MIDWIFERY/

NURSES/

PHYSICIANS/

exp HEALTH PERSONNEL/

PERINATAL DEATH/

ATTITUDE/

INTERVIEWS/

QUESTIONNAIRES/

FOCUS GROUPS/

QUALITATIVE STUDIES/

SURVEYS/

BNI:

FAMILY/

exp MIDWIFERY/

NURSES: ENROLLED/

STAFF NURSES/

PERINATAL AND NEONATAL MORTALITY/

exp DEATH: ATTITUDES

exp PATIENTS: ATTITUDES AND PERCEPTIONS

exp STAFF: ATTITUDES

INTERVIEWS AND INTERVIEWING/

ABORTION: SPONTANEOUS/

PsycINFO:

PARENTS/

FATHERS/

MOTHERS/

FAMILY/

HUMAN FEMALES/

HUMAN MALES/

MIDWIFERY/

NURSES/

PHYSICIANS/

OBSTETRICIANS/

HEALTH PERSONNEL/

ATTITUDES/

INTERVIEWS/

QUESTIONNAIRES/

QUALITATIVE RESEARCH/

SURVEYS/

SPONTANEOUS ABORTION/

AMED:

exp PARENTS/

FAMILY/

WOMEN/

MEN/

MIDWIFERY/

NURSES/

PHYSICIANS/

HEALTH PERSONNEL/

FETAL DEATH/

ATTITUDE/

INTERVIEWS/

QUESTIONNAIRES/
