# Supplementary material for: Systematic review to understand and improve care after stillbirth: a review of parents’ and healthcare professionals’ experiences
Source: BMC Pregnancy Childbirth. 2016 Jan 25;16:16. doi: 10.1186/s12884-016-0806-2 (PMC4727309; doi:10.1186/s12884-016-0806-2)
Supplement: Additional file 2: — Data collection form. (PDF 322 kb) [file 12884_2016_806_MOESM2_ESM.pdf]

**Title**	
**Author**	
**Citation**	
**Publication type**	

**Reader** (research team)	

*** Sample**	Population	
	Sample size	
	Geographical location	
	Method of recruitment	
	Time of involvement in study relative to the event	
	Period of data collection	
	Hypothesis/Research aims	
	Inclusion criteria	
	Exclusion criteria	
**Phenomenon of Interest**		
**Design**	Research type quant/qual/mixed	
	Method of data collection (detailed ie, who, when, where, how, tools used, how long)	
	Method of analysis (content/thematic etc)	
	Theoretical Framework	Postpositivist/ Constructivist/ Advocacy/ Pragmatic/ Not Described
	Interpretive communities	Feminist/ Postmodernist/ Critical theory/ Not Described
**Evaluation/ Measure of Outcome**		
**Techniques to reduce bias**	Multiple researchers	
	External qualitative researchers	
	Validation of transcripts by participants	
	Involvement of lay people in analysis	

** SPIDER tool[1] explanation*
Sample – Deemed more appropriate terminology than population/problem as smaller groups of participants tend to be used in qualitative research
Phenomenon of interest – As an intervention/exposure is not always evident in qualitative research questions this term is used to understand the how and why of certain behaviours, decisions and individual experiences.
Design – The theoretical framework used in qualitative research will determine the research method used. Details of the study design will help make decisions about the robustness of the study and analysis. In addition this might increase the detection of qualitative studies with unstructured titles and abstracts on databases.
Evaluation – Term deemed mores suitable to describe qualitative outcome measures which may be unobservable/subjective e.g. attitudes and views
Research type – qualitative, quantitative and mixed method

Findings:

	Extracted Teachable/Correctable Findings	Frequency of finding in the sample	Other indication of the importance of finding as identified by authors	Page & line
**Diagnosis**				
**Delivery**				
**Post mortem**				
**Support & follow-up**				

Evaluation by Reader:

**Quality of reporting**	
**Consistency between aims, methods and results**	

Summaries:

Section	Issue raised/discussed	Paper cited
**Introduction**		
**Findings/discussion**		

** SPIDER tool[1] explanation*
Sample – Deemed more appropriate terminology than population/problem as smaller groups of participants tend to be used in qualitative research
Phenomenon of interest – As an intervention/exposure is not always evident in qualitative research questions this term is used to understand the how and why of certain behaviours, decisions and individual experiences.
Design – The theoretical framework used in qualitative research will determine the research method used. Details of the study design will help make decisions about the robustness of the study and analysis. In addition this might increase the detection of qualitative studies with unstructured titles and abstracts on databases.
Evaluation – Term deemed mores suitable to describe qualitative outcome measures which may be unobservable/subjective e.g. attitudes and views
Research type – qualitative, quantitative and mixed method
